# The Hospital Pageant

**Published:** 1921-05-14

**Authors:** 


### THE HOSPITAL PAGEANT.

To use a homely phrase, the Hospital Pageant proposed in our leading article last week has "caught on." It would not be possible to publish or even quote from a tithe of the large number of letters we have received from hospital secretaries and others interested. It is very gratifying to us that the idea should have attracted so much enthusiasm and unanimity, for there is but one verdict on the project, and that is that it would be, as we suggested, a valuable part of a scheme of collective publicity and propaganda on behalf of the voluntary hospitals. The issues of how, when, and where such a pageant should be held are, of course, questions upon which opinions must necessarily differ; these points can only be determined by consultation amongst those who are interested, and if there is anything that this Journal can possibly do in the way of providing a central meeting-place or otherwise to help bring about such a consultation it will gladly be done.

The remarks of Mr. P. A. Inman, Secretary of the Hospital Saturday Fund, which will be found on page 108, will be read with much interest. Mr. Inman calls to mind the important pageant which was held in Dublin in 1913, when the whole history of nursing was shown from the earliest times until that date. Much has happened in respect of nursing and of hospitals since 1913, and what our hospitals, and the services they stand for, did during the Great War would provide an important inspiration of the proposed super-demonstration.

When we made our suggestion we felt that there was even more in it than could be pictured at the moment, and we are sure that, when the busy brains of our principal hospital workers get together, an event will be arranged which will do as much to educate the people of London to the hospital idea as has any other happening within the memory of the present generation. Nor is London alone interested in the venture; valuable experience will be gained which may very well be utilised in each of the great industrial centres of the land to gain new sympathisers and supporters to the cause of the provincial hospitals.

